# A Meta Analysis on Risks of Adverse Pregnancy Outcomes in *Toxoplasma gondii* Infection

**DOI:** 10.1371/journal.pone.0097775

**Published:** 2014-05-15

**Authors:** Xue-Lan Li, Hai-Xia Wei, Hao Zhang, Hong-Juan Peng, David S. Lindsay

**Affiliations:** 1 Department of Pathogen Biology, School of Public Health and Tropical Medicine, Southern Medical University, Guangzhou, Guangdong, the People's Republic of China; 2 Department of Infectious Diseases and Hepatology Unit, Nanfang Hospital attached to Southern Medical University, Guangzhou, Guangdong, and the People's Republic of China,; 3 Department of Biomedical Sciences & Pathobiology, VA-MD Regional College of Veterinary Medicine, Virginia Tech, Duck Pond Drive, Blacksburg, Virginia, United States of America; Technion-Israel Institute of Technology Haifa, Israel

## Abstract

**Objective:**

Quantified risks of congenital *Toxoplasma gondii* infection and abnormal pregnancy outcomes following primary maternal infection were evaluated with meta- analysis based on published studies.

**Methods:**

The related literatures were searched in multiple literature databases regardless of languages. Odds ratio (OR) and 95% confidence interval (CI) were used to evaluate the risks of vertical transmission of *Toxoplasma gondii* and abnormal pregnancy outcomes following primary maternal infection with meta-analysis.

**Results:**

53 of the 2632 searched literatures were included in our analysis. The incidence of abnormal pregnancy outcomes in *T. gondii* infected pregnant women (infected group) was significantly higher than that in the uninfected pregnant women (control group) (OR = 5.10; 95% CI, 3.85–6.75). *Toxoplasma gondii* infection rate in the abnormal-pregnancy-outcome group was significantly higher than in the normal-pregnancy group (OR = 3.71; 95% CI, 3.31–4.15). The pooled rate of vertical transmission was 20% (95% CI, 15%–26%) in maternal infection of *T. gondii*. The incidences of vertical transmission in women who were infected in the first, second or third trimester of pregnancy were 5% (95%CI, 2%–16%), 13% (95%CI, 7%–23%), and 32% (95%CI, 24%–41%), respectively. The rates of vertical transmission in women who were treated with spiramycin-only, PSF (pyrimethamine + sulfadiazine + folinic acid) or PS (pyrimethamine + sulfadiazine) combined with spiramycin, or other untypical treatments were 13% (95%CI, 7%–22%), 13%(95%CI, 7%–25%), and 24%(95%CI, 18%–32%), respectively.

**Conclusions:**

*Toxoplasma gondii* infection can result in adverse pregnancy outcomes in pregnant women. The pooled rate of vertical transmission was 20% in maternal infection and the incidences of vertical transmission increased in the first, second or third trimester of pregnancy. The pooled rates of transmission in groups treated with spiramycin-only, PSF or PS combined with spiramycin, or other untypical treatments were not significantly different.

## Introduction

*Toxoplasma gondii* is an intracellular protozoan parasite which is highly prevalent in humans and animals [1]. A wide variety of warm-blooded animals, including humans, can serve as the intermediate hosts of *T. gondii*, but its definitive host is limited to domestic cats and other felids [1], [2]. People become infected by ingestion of *T. gondii* tissue cysts in infected meat or by ingestion of infective oocysts shed by cats in contaminated food or water [3]. Primary infection of *T. gondii* in pregnant women can cause vertical transmission of the parasite and result in miscarriage, stillbirth, premature birth, malformations and other adverse pregnancy outcomes. Children with congenital toxoplasmosis may exhibit clinical signs of hydrocephalus, mental retardation, eye disease and other severe sequelae [4], [5]. Currently, congenital toxoplasmosis is believed to be the second most common seen fetal intrauterine infection [6]. Additionally, according to Torgerson and Mastroiacovo's study, the global annual prevalence of congenital toxoplasmosis was estimated to be 190 100 cases (95% confidence interval, CI: 179 300–206 300), which means the global burden of congenital toxoplasmosis was 1.20 million disability-adjusted life years (DALYs) (95% CI: 0.76–1.90) [7]. Hence, the poor health condition of children with congenital toxoplasmosis contributes to the heavy global health burden of children.

Women are usually symptomless when they acquire *T. gondii* infection in pregnancy. If maternal infection is detected, the mother usually receives treatment for toxoplasmosis and the fetus will face the risk of congenital infection. For treatment of *T. gondii* infection in pregnant women, the most commonly used drug is spiramycin because it can be absorbed efficiently and has little side effects to the fetus [8]. It is generally recommended to treat *Toxoplasma* infection with spiramycin in early trimesters, then change to PSF in the later trimesters [9].

Several studies have investigated the relationship between *T. gondii* infection and adverse pregnancy outcomes and the vertical transmission rate of *T. gondii,* but the parameters and methods used varied greatly in these studies. Because this is an extremely important health care issue, we used meta-analysis to evaluate the risks of vertical transmission and abnormal pregnancy outcomes in women experiencing primary infection with *T*. gondii during pregnancy.

## Materials and Methods

### Search strategy

Our study was performed according to the recommendations of the PRISMA Statement [10], which is available in (Checklist S1). We searched Pubmed, Embase, Google scholar, ScienceDirect, and CNKI database, Chongqing VIP database, Wanfang academic journal full-text database for papers published up to May 2013. Studies were identified using combinations of the following search terms regardless of languages: “*Toxoplasma* OR *gondii* OR toxoplasmosis” AND “pregnancy infection” AND “adverse pregnancy outcome OR abortion OR stillbirth OR abnormality OR fetal growth restriction OR FGR OR intrauterine growth retardation OR IUGR”.

### Literature citation inclusion and exclusion criteria

The literature citations were screened according to the following criteria. Inclusion criteria: (i) a case-control or cohort study or a survey with cases collected from clinical notes that related to our theme; (ii) the women in the control group were non-Toxoplasma-infected pregnant women and they were located in the same area as the women in the case group; (iii) the diagnosis of maternal T. gondii infection was based on seroconversion, parasite observation from cell culture or mouse ascites after inoculation of maternal blood, or PCR test of parasite DNA during gestation; (iv) the diagnosis of congenital *Toxoplasma* infection met one of the following standards: A. persistence of specific IgG in the child beyond 12 months or reappearance of IgG antibodies after cessation of antibiotic therapy, B. *Toxoplasma* specific IgM and/or IgA in cord blood and/or in neonatal blood (the purity of fetal blood was ascertained or the positive results were confirmed at least 7–10 days later), C. presence of parasite in amniotic fluid, placenta or fetal blood confirmed by inoculation to mice ascites, cell culture, or by PCR test. Literatures were excluded in the studies if (i) the paper was a review or a descriptive study; (ii) its subjects were not human beings but animals; (iii) the data was duplicate or the study only presented the final result without the raw data; (iv) the sample contained less than 40 participants or the number of participants in different groups was less than 10.

### Data extraction

The following information was extracted from each study: first author, publication year, location of the study, demographic characteristics, the number of cases and controls, diagnostic methods of cases, treatment regimes of the infected women, pregnancy outcomes, and gestational age of infection. In some studies, not all of the data were extracted because a portion of the data had already been reported. And for the republished studies, only the most complete or recent study was included. Two reviewers independently collected the data and reached a consensus after a discussion on the literatures which were controversial.

### Statistical analysis

The risk of *T. gondii* infection and various adverse pregnancy outcomes was estimated by odds ratio (OR) with the corresponding 95% confidence interval (95%CI). The pooled proportion of vertical transmission of toxoplasmosis with the corresponding 95%CI was calculated as well. It was considered statistically significant when P<0.05. In the forest plots, OR>1 represented a risk effect and OR<1 represented a protective effect. Statistical heterogeneity of results was appraised using a χ^2^-based Q test and I^2^ statistic [11]. Only when P>0.10 and I^2^<50% was the heterogeneity considered not significant. The fixed-effects model was used when literature heterogeneity not existed; otherwise, the random-effects model was employed. Sensitivity analysis was conducted by modification of the inclusion criteria of this meta-analysis. The pooled proportion of vertical transmission of toxoplasmosis was calculated by Meta-Analysis Beta 3.13 software (Tufts Medical Center, Boston, MA). The other analyses were conducted using Stata software version 11.0 (Stata Corporation, College Station, TX, USA) and the publication bias was considered significant when P value was less than 0.05 in either Begg's test or Egger's test [12].

## Results

### Studies characteristics

**Figure 1:**
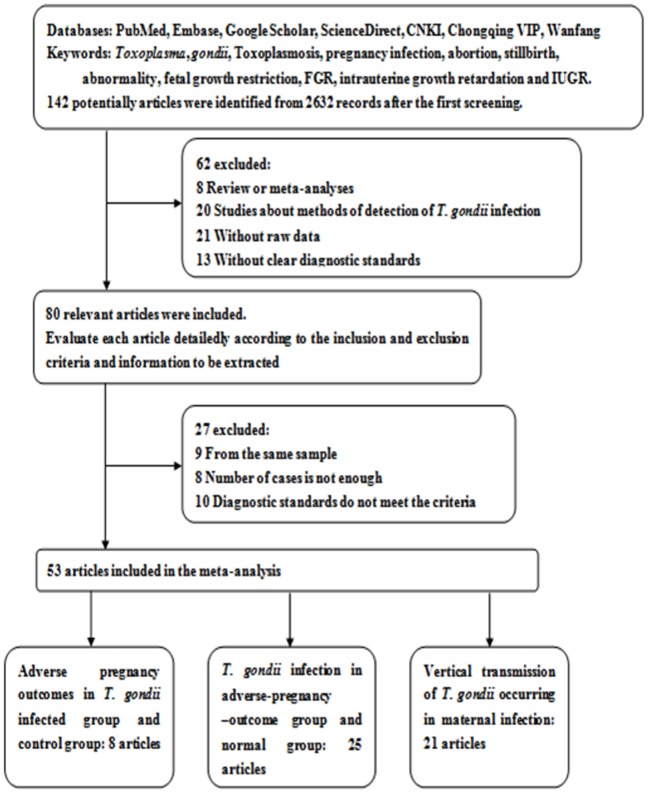
Flow diagram of the selection of the studies.

From the 2632 searched literatures, 53 were included and the results from these literatures were weighted [13]–[64],[74], including 8 studies about adverse pregnancy outcomes when the mother was infected with *T. gondii* and with control groups for each [13]–[20], 25 studies about infection rate in adverse outcomes and normal groups [21]–[44], [74], 21 studies about vertical transmission of the parasite [15], [45]–[64] (Figure 1). Further, 7 papers provided the detailed information about the gestation age when the woman was infected [46], [48], [49], [55], [58], [61], [64]. Additionally, two papers involved mothers that gave birth to twins [51], [53]. Some women received prenatal treatment in some studies [45], [47], [48], [50], [51], [53]–[55], [58], [59], [61], [64]. Details about the first author, published year, area, diagnostic standard, number of cases and controls and treatment regimes in each literature were listed in Tables 1, 2, 3 and 4.

**Table 1:** Studies about abnormal pregnancy outcomes in *T.gondii* infected groups and control groups.

First author	Year	Area	Cases/Controls^#^	Diagnosis of Maternal Infection	Abortion*	Premature Birth*	Fetal Anomaly*	FGR*	Stillbirth*	Reference
Su CK	2002	Guangxi	64/932	Positive IgM	-	0.06/0.02	0.08/0.01	-	0.03/0.01	[13]
Wen LZ	2003	East China	95/117	Positive IgM	0.13/0.03	0.04/0.02	0.03/0.01	0.04/0.02	0.05/0.01	[14]
Liu J	2004	Shanxi	76/986	Positive IgM and/or PCR	0.11/0.02	0.04/0.05	-	0.05/0.01	0.07/0.01	[15]
Yan Q	2006	Guangdong	64/932	Positive IgM and PCR	-	0.06/0.02	0.05/0.01	-	0.13/0.01	[16]
Yuan WY	2009	Hebei	325/147	Positive IgM	0.07/0.02	0.09/0.01	0.07/0.02	-	0.06/0.01	[17]
Suo QL	2011	Hubei	775/629	Positive IgM	0.07/0.01	0.02/0.01	0.03/0.01	0.03/0.01	0.03/0.01	[18]
Wang J	2011	Liaoning	149/5537	Positive IgM	0.09/0.01	-	0.08/0.01	-	0.03/0.01	[19]
Fang L	2012	Heilongjiang	273/496	Positive IgM	0.18/0.02	0.29/0.04	-	-	-	[20]

**Notes**:^ #^Cases, *Toxoplasma*-infected pregnant women, Controls, Non-infected pregnant women;* the data before and after the slash represent the rate of adverse pregnancy outcome in *T.gondii* infection groups and uninfected groups; - no statistics; “FGR”, fetal grown restriction.

**Table 2:** Studies about *T.gondii* infection rate in abnormal pregnancy and normal pregnancy.

First author	Year	Area	Diagnosis of Maternal Infection	Cases/Controls^#^	Infection rate^*^	Reference
Sahwi SY	1995	Bristol	Positive IgM and/or IgA	100/40	0.19/0.08	[42]
Moyo SR	1995	Zimbabwe	Positive culture	104/96	0.36/0.13	[41]
Zhang Y	2002	Tianjing	Positive PCR	1135/7141	0.01/0.00	[21]
Yang QF	2003	Guizhou	Positive IgM	86/100	0.07/0.02	[22]
Laila N	2004	Grenoble	Positive PCR	148/100	0.14/0	[44]
Cao MG	2004	Shandong	Positive IgM	1546/3568	0.09/0.01	[23]
Hu CM	2004	Guangdong	Positive IgM	101/1282	0.15/0.08	[25]
Chen HM	2004	Hubei	Positive IgM	476/562	0.13/0.05	[24]
Wei SZ	2005	Fujian	Positive IgM	117/1695	0.13/0.05	[26]
Yang AJ	2005	Shandong	Positive PCR	380/152	0.21/0.04	[27]
Ye HZ	2005	Guangdong	Positive IgM	93/944	0.03/0.00	[28]
Chen MR	2006	Shandong	Positive IgM	1546/3568	0.15/0.03	[29]
Li BY	2006	Guangdong	Positive IgM	48/48	0.33/0.04	[30]
Xie DC	2006	Guangxi	Positive IgM	502/400	0.14/0.06	[31]
Chen XJ	2007	Jilin	Positive IgM	200/1805	0.24/0.07	[32]
Guo EP	2008	Hubei	Positive IgM	71/819	0.14/0.03	[33]
Zhan HY	2008	Jiangsu	Positive IgM	197/200	0.10/0.02	[34]
Weng H	2009	Zhejiang	Positive IgM	89/102	0.20/0.05	[35]
Janak K	2011	Lucknow	Positive IgM	60/29	0.08/0	[43]
Long C	2011	Hubei	Positive IgM	402/3449	0.03/0.00	[36]
Qiu JZ	2011	Hunan	Positive IgM	193/512	0.06/0.01	[37]
Wang JY	2011	Hebei	Positive IgM	102/102	0.13/0.12	[38]
Wang KB	2012	Sichuan	Positive IgM	126/1430	0.13/0.04	[40]
Munmun DS	2012	India	Positive IgM	105/105	0.22/0.03	[39]
Aljumaili ZKM	2013	Iraq	Positive IgM	293/245	0.02/0	[74]

**Notes**:^ #^ the data before and after the slash represent the sample in abnormal pregnancy group and normal pregnancy group;* the data before and after the slash represent the *T.gondii* infection rate in abnormal pregnancy group and normal pregnancy group.

**Table 3:** Studies about the rate of vertical transmission when mother got infected in pregnancy.

First author	Year	Area	Diagnostic Standards		Rate^*^	Reference
			Mother	Baby^#^		
Berrebi A	1994	Toulouse	Seroconversion	Positive IgM, PCR or culture, clinical signs	0.17	[56]
Pratlong F	1994	Montpellier	Seroconversion, high-titre IgG with IgM	Positive IgM and IgA, culture	0.11	[50]
Hohlfeld P	1994	Paris	Seroconversion	Positive IgM, PCR or culture	0.07	[54]
Dar FK	1997	UAE	High-titre IgM	Positive IgM	0.38	[52]
Jenum A	1998	Norway	Seroconversion	Persistent IgG beyond 12 months, positive PCR or culture	0.23	[64]
Gratzl R	1998	Austria	Seroconversion, high-titre IgG and IgM	Persistent IgG beyond 12 months, positive PCR	0.22	[51]
Foulon W	1999	France	Seroconversion	Persistent IgG beyond 12 months, reappearance of IgG after therapy	0.44	[57]
Robert-Gangneux F	1999	Paris	Seroconversion	Persistent IgG beyond 12 months, positive PCR or culture	0.25	[59]
Naessens A	1999	America	Seroconversion	Persistent IgG beyond 12 months, reappearance of IgG after therapy	0.32	[57]
Lebech M	1999	Denmark	Seroconversion	Persistent IgG beyond 12 months, positive IgM and/or IgA	0.19	[49]
Gilbert R	2001	EUR,Austria	Seroconversion	Persistent IgG beyond 12 months, positive PCR or culture	0.24	[62]
Antsaklis A	2002	Athens	Seroconversion	Positive IgM, PCR or culture	0.19	[60]
Logar J	2002	Ljubljana	High-titre IgG, high-titre IgM and/or IgA	Positive IgM and IgA	0.11	[61]
Ricci M	2003	Italy	Seroconversion, high-titre IgG and IgM	Persistent IgG beyond 12 months	0.11	[55]
Mombro M	2003	Italy	Seroconversion, positive cultures	Persistent IgG beyond 12 months, reappearance of IgG after therapy, specific IgM and/or IgA	0.22	[46]
Liu J	2004	China	Positive PCR, high-titre IgM	Positive PCR	0.37	[15]
Di Carlo P	2005	Italy	Seroconversion	Persistent IgG beyond 12 months, positive PCR	0.19	[58]
Buffolano W	2005	Campania	Seroconversion	Persistent IgG beyond 12 months	0.34	[63]
Berrébi A	2010	Toulouse	Seroconversion	Persistent IgG beyond 12 months	0.17	[53]
Hotop A	2012	Germany	Seroconversion	Persistent IgG beyond 12 months, positive PCR	0.05	[48]
Wallon M	2013	Lyon	Seroconversion, high-titre IgG and IgM	Persistent IgG beyond 12 months, positive culture	0.25	[45]

**Notes**:^#^ For the positive IgM/IgA results, the purity of fetal blood was ascertained or the positive results were confirmed at least 7-10 days later; ^*^ Rate stands for vertical transmission rate caused by *T.gondii* infection.

**Table 4:** Studies about the rate of vertical transmission when infected mother got treatment in pregnancy.

First Author	Year	Treatment	Infected Mother	Infected Baby	Rate	Reference
Pratlong F	1994	Spir-only	190	20	0.11	[50]
Hohlfeld P	1994	Spir-only	2632	194	0.07	[54]
Gratzl R	1998	Spir-only	12	1	0.08	[51]
		PSF/Spir	37	10	0.27	[51]
Jenum A	1998	PS/Spir	47	11	0.23	[64]
Robert-Gangneux F	1999	Spir-only	110	27	0.25	[59]
Naessens A	1999	Others^1^	294	93	0.32	[47]
Logar J	2002	PSF/Spir	100	11	0.11	[61]
Ricci M	2003	PSF/Spir	141	16	0.11	[55]
Buffolano W	2005	Spir-only	74	12	0.16	[58]
Berrébi A	2010	Others^2^	666	112	0.17	[53]
Hotop A	2012	PSF/Spir	685	33	0.05	[48]
Wallon M	2013	Others^3^	2048	513	0.25	[45]

**Notes**: Spir-only, spiramycin only; PS/Spir, PS in combination with spiramycin; PSF/Spir, PSF in combination with spiramycin; Others, other untypical treatment, ^1^ only 75% of infected women were administered to antibiotic treatment, the rest were conducted with other medicine; ^2^ 80% of infected women were administered to pyrimethamine-sulfadoxine, 20% were taken with spiramycin; ^3^ PS alternated every 3 weeks with spiramycin before 1996, and then PS was taken continually.

### Quantitative synthesis and heterogeneity analysis

**Figure 2:**
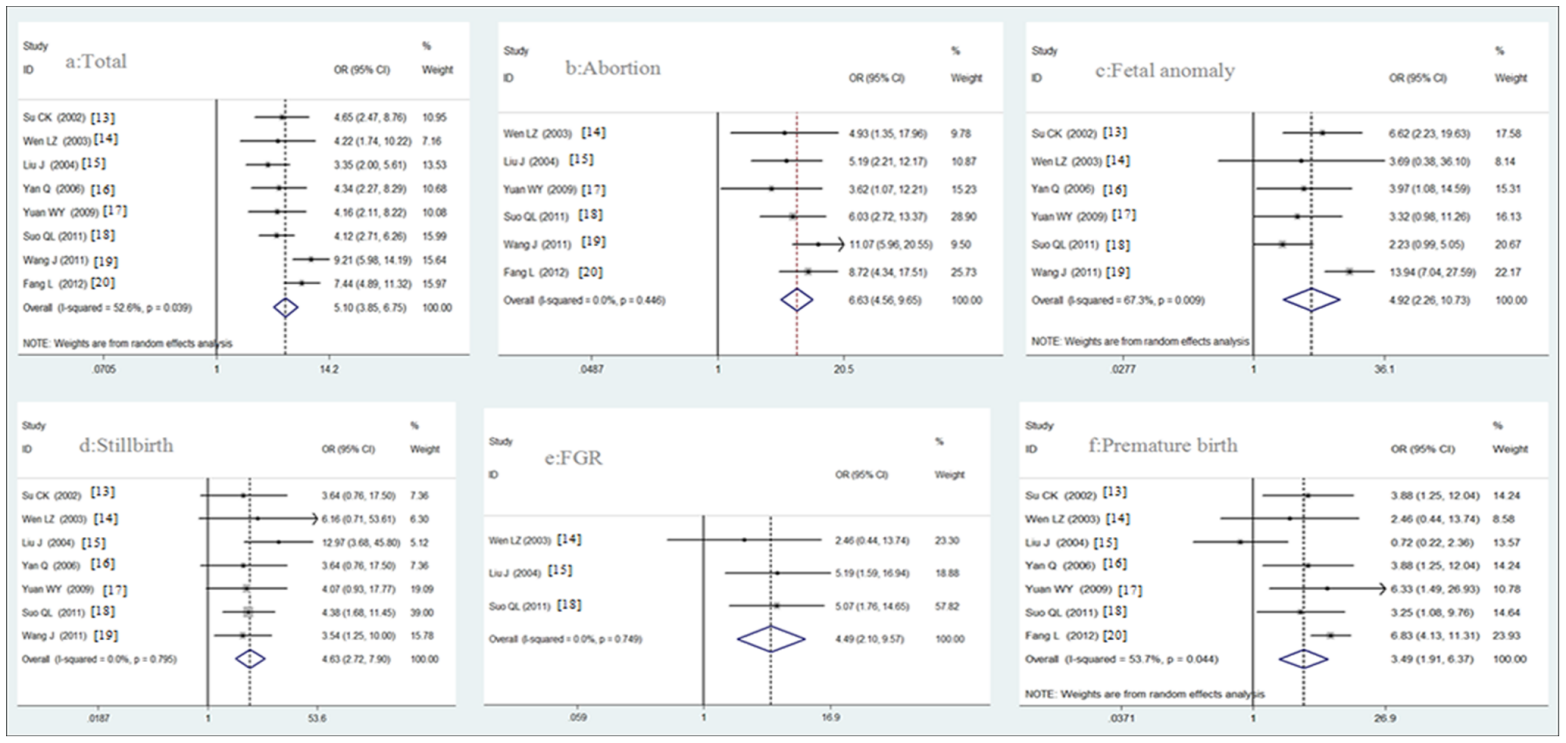
Forest plot of the relationship between *T.gondii* infection and adverse pregnancy outcomes. a, The odds ratio of the total abnormal pregnancy chance between *Toxoplasma* infected and uninfected pregnant women; b-f, The odds ratio of the different abnormal pregnancy outcomes between *Toxoplasma* infected and uninfected pregnant women. Scale: for value of odds ratio.

**1. Comparison of the abnormal pregnancy chances between** ***T. gondii*** **infected and uninfected pregnant women.** The prevalence of abnormal pregnancy outcomes in *T. gondii* infected pregnant women (infected group) was significantly higher than in the uninfected pregnant women (control group) (P<0.05); the OR was 5.10 (95% CI, 3.85–6.75) analyzed with the random-effects model. Among these abnormal pregnancy outcomes, the prevalence of abortion, fetal anomaly, stillbirth, FGR (fetal growth restriction), and premature birth were all significantly higher in the infected group than that in the control group (P<0.05), with OR and 95% CI of 6.63 (4.56–9.65), 4.92 (2.26–10.73), 4.63 (2.72–7.90), 4.49 (2.10–9.57), and 3.49 (1.91–6.37), respectively (Figure 2). The detail analysis results were shown in Table 5.

**Table 5:** Analysis results of the relationship between maternal *T.gondii* infection and adverse pregnancy outcomes.

Outcomes	Test of risk		Test of heterogeneity			Model	Reference
	Odds Ratio (95%CI)	P	Q	P	I^2^ (%)		
Abortion	6.63 (4.56 to 9.65)	p<0.0001	4.76	0.04	<0.01	Fixed-effects model	[14], [15], [17]–[20]
Fetal anomaly	4.92 (2.26 to 10.73)	p<0.0001	15.30	0.01	67.3	Random-effects model	[13], [14], [16]–[19]
Stillbirth	4.63 (2.72 to 7.90)	p<0.0001	3.11	0.80	<0.01	Fixed-effects model	[13]–[19]
FGR	4.49 (2.10 to 9.57)	p<0.0001	0.58	0.75	<0.01	Fixed-effects model	[14], [15], [18]
Premature birth	3.49 (1.91 to 6.37)	p<0.0001	12.95	0.04	53.7	Random-effects model	[13]–
Total	5.10 (3.85 to 6.75)	p<0.0001	14.76	0.04	52.6	Random-effects model	[13]–[20]

**Figure 3:**
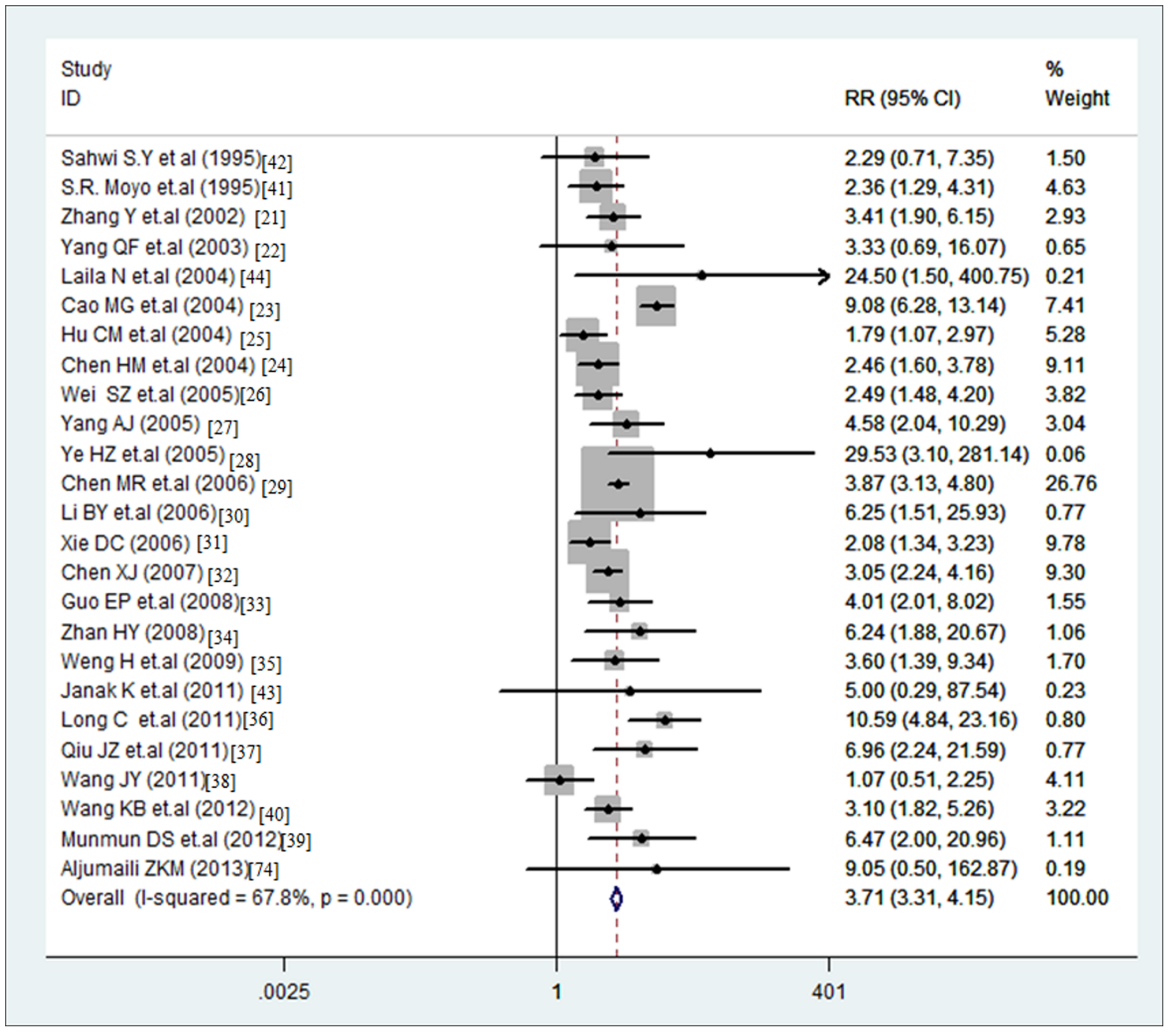
Odds ratio of *Toxoplasma* infection rate between abnormal pregnancy and normal pregnancy. Scale: for value of odds ratio.

**2. Comparison of** ***T. gondii*** **infection rate between abnormal pregnancy and normal pregnancy groups.** The *Toxoplasma gondii* infection rate of abnormal-pregnancy-outcome group was significantly higher than in the normal-pregnancy group (P<0.05), with an OR of 3.71 (95% CI, 3.31–4.15) analyzed with the random-effects model (Q = 74.62, p<0.0001, I^2^ = 67.8%) (Figure 3).

**Figure 4:**
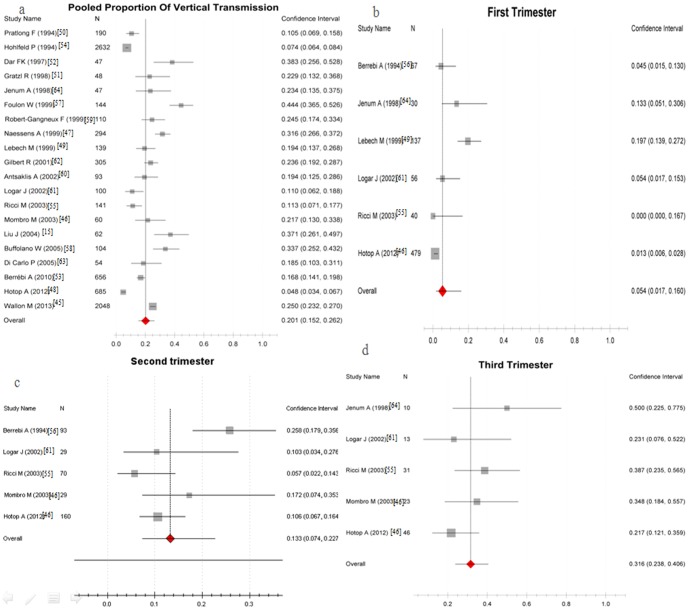
Proportion of congenital toxoplasmosis happening by mother infection. a, The rate of vertical transmission when mother got infected in pregnancy; b-d, The rate of vertical transmission in different pregnancy trimester. Scale: incidence of congenital toxoplasmosis.

**3. Chance of congenital** ***T. gondii*** **transmission occurring in maternal infection.** The rate of congenital transmission of *T. gondii* in maternal infection was 20% (95% CI, 15%–26%), which suggested that about 20% of infected mothers would transmit the parasite to fetus. The rate of vertical transmission in women who were infected in the first, second or third trimesters of pregnancy were 5% (95%CI, 2%–16%), 13% (95%CI, 7%–23%), and 32% (95%CI, 24%–41%), respectively (Figure 4). The detailed analysis results are shown in Table 6.

**Table 6:** Analysis results of the rate of vertical transmission when mother got infected in different trimester.

Time	Test of risk		Test of heterogeneity			Model	Reference
	Pooled Proportion (95%CI)	P	Q	P	I^2^ (%)		
First trimester	0.05 (0.02 to 0.16)	<0.0001	0.979	<0.001	47.2	Random-effects model	[48], [49], [55], [56], [61], [64]
Second trimester	0.13 (0.07 to 0.28)	<0.0001	0.939	0.004	42.5	Random-effects model	[46], [48], [55], [56], [61]
Third trimester	0.32 (0.24 to 0.41)	<0.0001	0.827	0.237	13.9	Fixed-effects model	[46], [48], [55], [61], [64]
Total pregnancy	0.20 (0.15 to 0.26)	<0.0001	0.998	<0.001	49.0	Random-effects model	[15], [45]–[64]

**Figure 5:**
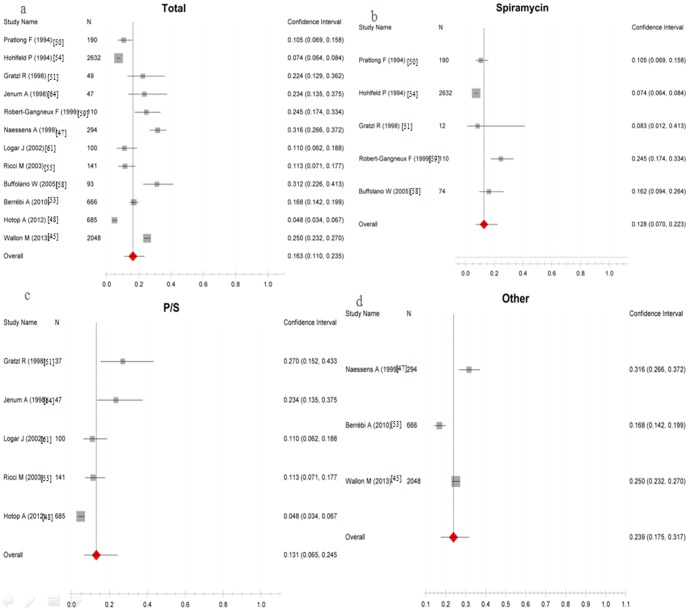
Proportion of congenital toxoplasmosis happening when infected mother received prenatal treatment. a, The total rate of vertical transmission when mother received treatment; b-d, The rate of vertical transmission when mother received different treatment regimes. Scale: incidence of congenital toxoplasmosis.

The pooled rate of congenital transmission of *T. gondii* occurring in women who received treatment was 16% (95% CI, 11%–24%), which suggested that about 16% of treated infected mothers would transmit the parasite to fetus. The rate of vertical transmission in women who received Spiramycin-only, PSF or PS in combination with spiramycin, or other untypical treatment were 13%(95%CI, 7%–22%), 13%(95%CI, 7%–25%), and 24%(95%CI, 18%–32%), respectively (Figure 5). The detailed analysis results are shown in Table 7.

**Table 7:** Analysis results of the vertical transmission rate when infected mother got treatment in pregnancy.

Treatment	Test of risk		Test of heterogeneity			Model	Reference
	Pooled Proportion (95%CI)	P	Q	P	I^2^ (%)		
Spir-only	0.128 (0.070 to 0.223)	<0.0001	0.977	<0.001	47.5	Random-effects model	[50], [51], [54], [58], [59]
P/S	0.131 (0.065 to 0.245)	<0.0001	0.975	<0.001	47.3	Random-effects model	[48], [51], [55], [61], [64]
Others	0.239(0.175 to 0.317)	<0.0001	0.967	<0.001	48.2	Random-effects model	[45], [47], [53]
Total	0.163 (0.110 to 0.235)	<0.0001	0.997	<0.001	49.3	Random-effects model	[45], [47], [48], [50], [51], [53]–[55], [58], [59], [61], [64]

**Notes**: Spir-only, spiramycin only; P/S, PS or PSF in combination with spiramycin; Others, other untypical treatment.

### Sensitivity analysis

A sensitivity analysis was conducted to ascertain whether modification of the inclusion criteria of this meta-analysis affected the final results. On the analysis of the association between *T. gondii* infection and the abnormal pregnancy outcomes, the sensitivity analysis was carried out by excluding one single study each time and limiting the meta-analysis to studies with sample size more than 100. All the results were not materially altered.

### Publication bias

**Figure 6:**
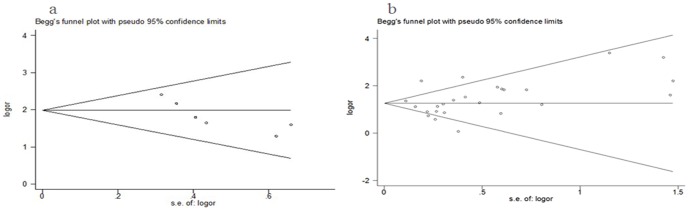
Funnel plot showing publication bias. a, in group of abortion and *T. gondii* infection; b, in group of infection rate in normal and abnormal pregnancy outcomes.

For prevalence of abortion between infected groups and uninfected groups and the *T. gondii* infection rate between abnormal pregnancy and normal pregnancy, the publication bias showed statistical significance (Begg's test, p = 0.060.0.059; Egger's test, p = 0.025,0.516) (Figure 6). In the other analysis, no publication bias was suggested.

## Discussion

Several studies have investigated the relationship between maternal infection with *T. gondii* and the adverse pregnancy outcomes including miscarriage, stillbirth, premature birth, and malformations. Our meta-analysis results confirmed this relationship and showed that miscarriage was the highest risk (OR = 6.63; 95% CI, 4.56–9.66) among the adverse pregnancy outcomes. Furthermore, a population-based study on the effects of congenital toxoplasmosis found out that infected babies were born or delivered earlier than uninfected babies, but the mechanism leading to a shorter length of gestation is unknown [65]. Additional studies are needed to determine whether adverse pregnancy outcomes after acquisition of *T. gondii* infection are related to a consequence of fetal infection or an effect of maternal infection. Additionally, mechanism of *T. gondii* causes placental inflammation and infects the fetus remains unknown.

Our study also showed that later infection during pregnancy was more likely to result in congenital infection, which was consistent with Dunn's and Foulon's studies, but the 30% vertical transmission rate in the third trimester of pregnancy in our meta analysis was much lower than that of 60% in their studies [57], [66], which possibly resulted from the small sample in their studies. Children with congenital *Toxoplasma* infection had more severe clinical symptoms when the mother acquired acute *T. gondii* infection during the first trimester than in the third trimester [54], [67], [68]. This may be due to the placental trophoblast, which is not conductive to the propagation of *T. gondii* and could prevent the parasite from crossing the placenta in early gestation [69]. But in later trimesters, the parasite is more likely to get through the placental barrier, so transmission is more frequent in later pregnancy than in earlier pregnancy. If the infection occurred in the first trimester, owing to the immature development and the low resistance of the fetus, the prevalence of sequelae may be higher than the infection happened in a later trimester [69], [70].

The rate of vertical transmission in women who were treated with spiramycin only, PSF or PS in combination with spiramycin, were 13% (95%CI, 7%–22%), and 13% (95%CI, 7%–25%), respectively. Comparing to Lebech M's study, the transmission rate of untreated pregnant women was 19% (95%CI, 13%–27%) [49], so we speculated that there was a low risk of vertical transmission in treated women with *Toxoplasma* infection during pregnancy. However, the effect of the prenatal treatment remains vague as there was no clear evidence from biological studies that prenatal treatment would reduce the risk of congenital infection. To prove whether the treatment regimes have a significant impact on pregnancy outcome, a clinical study with a large sample and an untreated comparison group is needed.

To avoid unnecessary drug therapy and pregnancy termination, much effort had been put to find an effective, quick, safe and cheap method for prenatal diagnosis of maternal infection. Now it is available through PCR on amniotic fluid, which was confirmed to be the most reliable method with high sensitivity and high specificity [54], [71]. Moreover, in Austria, apart from the routine prenatal maternal *T. gondii* serology screening, the identification of *T. gondii* infection is significantly improved by the additional maternal and/or fetal serological testing at birth [72]. Many countries have adopted the prenatal screening program and it has been proved to be effective in France at reducing the rate of congenital infection [73].

In order to provide precise and updated information for *T. gondii* infected pregnant women with clinical counseling, this study adopted the strict diagnostic criteria to screen the cases in each literature citation. However, our meta-analysis still has several limitations. First, on analyses the risks of *T. gondii* infected women with abnormal pregnancy outcomes, most studies involved are from China owing to few equivalent foreign studies. Second, as only a few studies provided the exact gestation age at maternal infection, so the pooled vertical transmission rate of congenital toxoplasmosis was calculated based on the trimester of pregnancy rather than weeks. Third, the diagnostic methods of infected mother/fetal in different literature citations were not adopted uniformly, which may increase the source of the heterogeneity.

## Conclusions

This meta-analysis confirms the previous results that primary maternal infection of *T. gondii* during gestation plays a crucial role in adverse pregnancy outcomes. The incidences of abortion, fetal anomaly, stillbirth, fetal growth restriction, and premature birth were significantly higher in the infected group than in the control group, and showed in declining Odds Ratios. Reversely, *Toxoplasma gondii* infection rate in the abnormal-pregnancy-outcome group was significantly higher than in the normal-pregnancy group. The pooled rate of vertical transmission was 20% in maternal infection and the incidences of vertical transmission increased with the pregnancy time (the first, second or third trimester of pregnancy). Compared to the vertical transmission rate of 32% in the third trimester, the rate (5%) was much lower when the infection occurred in the first trimester. The pooled rate of vertical transmission in maternal infection received treatment was 16%, and the rates of treatment with spiramycin-only, PSF or PS combined with spiramycin, or other untypical treatments were not significantly different.

## Supporting Information

Checklist S1 **PRIMSA checklist of this meta-analysis.**
(DOC)
